# High-Throughput Sequencing Reveals *Cyclamen persicum* Mill. as a Natural Host for Fig Mosaic Virus

**DOI:** 10.3390/v10120684

**Published:** 2018-12-03

**Authors:** Toufic Elbeaino, Armelle Marais, Chantal Faure, Elisa Trioano, Thierry Candresse, Giuseppe Parrella

**Affiliations:** 1 Mediterranean Agronomic Institute of Bari (CIHEAM-IAMB), Via Ceglie 9, 70010 Valenzano, Italy; 2 UMR 1332 Biologie du Fruit et Pathologie, INRA, Université Bordeaux, CS 20032, 33882 Villenave d’Ornon CEDEX, France; armelle.marais-colombel@inra.fr (A.M.); chantal.faure@inra.fr (C.F.); thierry.candresse@inra.fr (T.C.); 3 Institute for Sustainable Plant Protection, National Research Council (CNR), Via Università 133, 80055 Portici, Italy; elisa.troiano@ipsp.cnr.it

**Keywords:** high-throughput sequencing, cyclamen, *Fig mosaic emaravirus*

## Abstract

In a search for viral infections, double-stranded RNA (dsRNA) were recovered from a diseased cyclamen (*Cyclamen persicum* Mill.) accession (Cic) and analyzed by high-throughput sequencing (HTS) technology. Analysis of the HTS data showed the presence of *Fig mosaic emaravirus* (FMV) in this accession. The complete sequences of six FMV-Cic RNA genomic segments were determined from the HTS data and using Sanger sequencing. All FMV-Cic RNA segments are similar in size to those of FMV from fig (FMV-Gr10), with the exception of RNA-6 that is one nucleotide longer. The occurrence of FMV in cyclamen was investigated through a small-scale survey, from which four plants (out of 18 tested) were found RT-PCR positive. To study sequence variations of cyclamen isolates of FMV, RT-PCR products generated through the amplification of the partially RNA-dependent RNA polymerase (RdRp, RNA-1), glycoprotein (GP, RNA-2), and nucleocapsid (NCP, RNA-3) genes were explored. The nucleotide sequence identities for cyclamen isolates ranged between 86% and 99% in RNA-1, 93% and 99% in RNA-2, and 98% and 99% in RNA-3, while lower identity levels were observed with the sequences of fig isolates. Based on the phylogenetic tree obtained with a 304-nt fragment of RNA3, all FMV isolates from cyclamens were assigned to a single cluster close to fig isolates from the Mediterranean. FMV was graft-transmitted to healthy cyclamens eliciting symptoms similar to those observed in the Cic accession, thus suggesting a causal role of FMV in the symptoms that prompted the investigation. This is the first report of FMV in a non-fig host, *Cyclamen persicum*, a finding that may help in the control of the mosaic and mosaic-like diseases of fig and cyclamen, respectively.

## 1. Introduction

Cyclamen (*Cyclamen persicum* Mill.) is a potted flowering plant originating from the eastern Mediterranean coast. It is of major significance in the horticultural trade. Many viruses infect it in nature, the best-known being tomato spotted wilt virus and impatiens necrotic spot virus (TSWV and INSV, respectively, genus *Orthotospovirus*), cucumber mosaic virus and tomato aspermy virus (CMV and TAV, respectively, genus *Cucumovirus*), tobacco mosaic virus (TMV, *Tobamovirus*), tobacco rattle virus (TRV, *Tobravirus*), and potato virus X (PVX, *Potexvirus*). All these viruses are associated with different types of symptoms, ranging from discoloration, yellowing, mottling, and necrosis to mosaics, so that their impact on trade is enormously negative.

**Figure 1:**
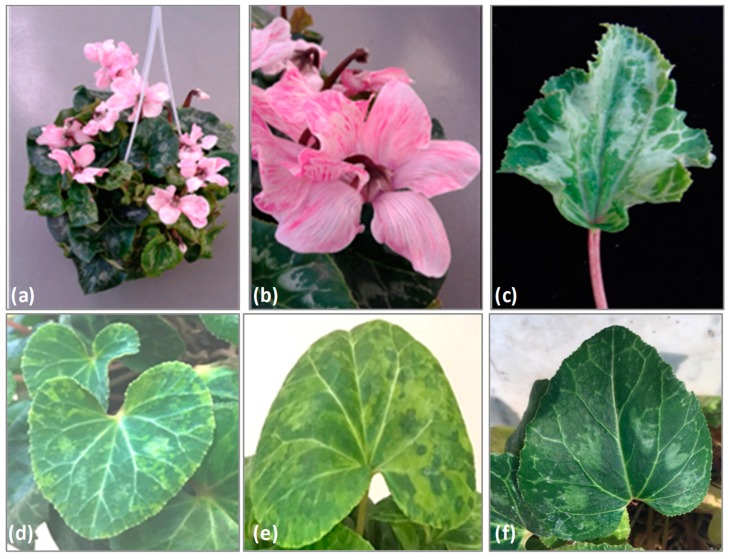
Symptoms observed on *Cyclamen persicum* (accession Cic) found infected with *Fig mosaic emaravirus* (FMV). (**a**) Cic-potted plant; (**b**) flowers showing color breaking and stripes; (**c**) Leaf with mottling, light yellowing and deformation; (**d**,**e**) leaves grafted onto Cic- and G-potted accessions showing mottling and light yellowing symptoms; (**f**) healthy leaf of cyclamen.

A diseased cyclamen plant (accession Cic), from the town of Ercolano (Campania region, South Italy), with virus-like symptoms (leaf mottling and yellowing, leaf deformation, flower breaking, (Figure 1)) resulted negative when assayed with specific RT-PCR tests against all aforementioned viruses. This outcome prompted an attempt at virus characterization using a high-throughput sequencing (HTS) approach. Unlike other technologies, this approach does not require any prior sequence information of the pathogen and has the potential to identify all viral agents infecting a sample. Bioinformatic analysis of the HTS data revealed the presence of *Fig mosaic emaravirus* (FMV), the etiological agent of the mosaic disease (MD) of fig [1], whose presence has never been reported before on cyclamen. Importantly, no fig tree was present in the vicinity of the nursery where Cic accession was found.

FMV belongs to the genus *Emaravirus* in the newly established family *Fimoviridae* [2]. Its genome consists of six single-stranded, negative-sense RNA segments [1,3,4], which respectively encode the RNA-dependent RNA polymerase (RdRp, RNA-1), a putative glycoprotein (GP, RNA-2), a putative nucleocapsid protein (NCP, RNA-3), a putative movement protein (MP, RNA-4), and two proteins with unknown functions (p5 and p6, respectively RNA-5 and RNA-6) [3,4]. This virus is transmitted in nature by the eriophyid mite vector *Aceria ficus* [4,5], but not by seed or by mechanical inoculation. The finding of FMV in a new host has necessitated a molecular, biological and epidemiological analysis of the FMV-cyclamen isolate(s), the results of which are reported here.

## 2. Materials and Methods

### 2.1. Plant Source Material

One cyclamen plant (denoted as Cic), collected in June 2016, in a farm located in the town of Ercolano (Campania region, South Italy), exhibiting symptoms consisting of stripes on flowers and light leaf yellowing and deformation (Figure 1) was analyzed by a double-stranded RNA (dsRNA)-based HTS approach (see below). Later on, 18 cyclamen plants, four of which exhibited symptoms similar to those observed in the original Cic plant, were collected from two nurseries in the Bari province (South Italy) and analyzed by various RT-PCR virus-specific assays.

### 2.2. Extraction of Double-Stranded RNAs, High-Throughput Sequencing, and Sequence Bioinformatics Analysis

Double-stranded RNAs (dsRNAs) were extracted from five grams of symptomatic leaves of the Cic accession according to Gentit et al. [6], and thoroughly purified as described in Candresse et al. [7]. The purified dsRNAs were randomly reverse-transcribed and submitted to Illumina sequencing in a multiplexed format as described in Candresse et al. [7]. After demultiplexing and quality trimming steps, reads were assembled using CLC Genomics Workbench 9 (http://www.clcbio.com). The obtained contigs were annotated by Blastn and BlastX analyses against the Genbank database. Scaffolds were finally assembled by mapping the FMV contigs and residual reads on the FMV reference genome. The complete sequences of the six FMV-Cic genome segments were fully reconstructed by Sanger sequencing of overlapping PCR fragments obtained using a battery of sense and antisense primers designed on de novo assembled contigs (Supplementary Table S1), together with those specific to amplifying the 5′ and 3′ termini of all FMV segments [1,4,8]. PCR amplifications were performed using the proofreading TaKaRa LaTaq polymerase (TaKaRa, Bio Inc., Shiga, Japan) under the following cycling conditions: initial denaturation at 94 °C for 4 min; 35 cycles of 94 °C for 30 s, 56 °C for 30 s, and 72 °C for 1 min s; final extension step at 72 °C for 10 min.

### 2.3. Extraction of Total Nucleic Acids and PCR Detection of Cyclamen-Infecting Viruses and Phytoplasmas

Samples consisted of fresh leaves from symptomatic and asymptomatic plants, from which the total nucleic acids (TNA) were extracted according to Foissac et al. [9]. In brief, 200 mg of tissue from leaf veins were homogenized in 1 mL grinding buffer (4.0 M guanidine thiocyanate, 0.2 M NaOAc pH 5.2, 25 mM EDTA, 1.0 M KOAc pH 5.0, and 2.5% w/v PVP-40). After centrifugation for 10 min at 13,000× *g*, the supernatant was mixed with 6 M sodium iodide and 0.15 M sodium sulphite, 150 µL ethanol and 40 µL silica particles in suspension (1 g mL^−1^ pH 2.0). After stripping by heat treatment in sterile water (70 °C for 3 min) and centrifugation for 3 min at 16,000× *g*, TNAs were recovered and stored at −20 °C until use.

TNA were reverse-transcribed using 0.5 µg random DNA hexanucleotide mixture (Roche Diagnostics, Mannheim, Germany) and 200 units of *Moloney murine leukaemia virus* (M-MLV) reverse transcriptase enzyme in a 20 µL reaction for 1 h at 42 °C, following the manufacturer’s instructions (Invitrogen, Waltham, MA, USA).

The presence of TSWV, INSV, CMV, TAV, TMV, TRV, and PVX in the collected samples was explored through virus-specific RT-PCR assays. PCR conditions and cycles are described in Mumford et al. [10,11], Adkins and Rosskopf [12], Grieco et al. [13], Peiman and Xie [14], Riga et al. [15], and Verma et al. [16]. For the detection and partial sequence characterization of FMV in cyclamen, three primers pairs which amplify respectively the partial RdRP, GP, and NCP genes of FMV were used according to [1,4].

The potential presence of phytoplasmas in the various cyclamen samples was also explored by two rounds of polymerase chain reaction assays (PCR and nested-PCR) for the amplification of the 16S rRNA gene, using primers R16F1/R16R0 [17,18] followed by primer pair R16F2n/R16R2 [19]. These PCR tests were performed on DNA extracted from fresh cyclamen leaves according to Ahrens and Seemüller [20].

### 2.4. Sequence Analyses

All PCR products were ligated in Strata-Clone™ PCR cloning vector pSCA (Stratagene, La Jolla, CA, USA), cloned into *Escherichia coli* DH5α cells, and finally custom sequenced (Eurofins Genomics, Ebersberg bei München, Germany). Multiple alignments with the distance matrix for both nucleotide and amino and phylogenetic tree reconstruction were performed using CLC Genomics Workbench v.3.6.5 on the obtained FMV sequences and on corresponding sequences available in the GenBank database (www.ncbi.nlm.nih.gov).

### 2.5. Grafting and Mechanical Transmission of FMV

Two FMV-infected cyclamen accessions (Cic and G) were used as virus source for mechanical transmission and grafting experiments. Tissues from symptomatic leaves were ground in 0.1 M phosphate buffer, pH 7.2, in the presence of 2.5% nicotine, and sap was mechanically inoculated using carborundum onto herbaceous hosts, i.e., *Nicotiana benthamiana*, *Nicotiana occidentalis*, *Nicotiana tabacum* cv. Samsun, *Chenopodium quinoa*, *Chenopodium amaranticolor*, *Cucumis sativus*, *Vigna unguiculata*, and *Phaseolus vulgaris* cv. La Victoire.

Stems with young leaves from a healthy cyclamen plant, which yielded RT-PCR negative results for the presence of cyclamen viruses (including FMV) and phytoplasmas, were grafted onto homologous organs of the Cic or of the G FMV-infected potted plants using clips. Five stems with leaves were grafted onto each accession.

## 3. Results and Discussion

### 3.1. HTS Data Analyses and Identification of FMV

High-throughput Illumina sequencing of randomly amplified cDNA prepared from purified dsRNAs extracted from the Cic cyclamen accession yielded a total of 875,401 high quality reads (average length of 160 nt). De novo assembly of these reads yielded 193 contigs of more than 400 nt, which were then annotated by BlastN and BlastX analysis against the Genbank database. Besides some contigs with relatively distant homologies to dsRNA viruses, only six contigs were identified as viral with high Blast e-values. These contigs corresponded to genomic RNAs 3 to 6 of FMV (two contigs each for segments 3 and 5). Further analysis, by mapping the Illumina reads against the FMV reference genome (NC029562-8), allowed to identify a total of 4302 reads (0.49% of total reads) mapping to the genomic RNAs of FMV.

The sequences of the six RNA segments of the Cic cyclamen isolate of FMV were almost completely re-sequenced using Sanger sequencing of RT-PCR products obtained using a battery of sense and antisense specific primers (Supplementary Table S1) designed on HTS-generated FMV contigs or from the FMV reference sequences. The obtained complete sequences have been deposited in Genbank under the accession numbers: LT978305-LT978307 and MG813799-MG813801.

Sequence comparisons show that five RNA segments of FMV-Cic are similar in length to those of the reference FMV from fig (isolate Gr10), whereas RNA6 is longer by one nucleotide (1213 nt). All segments share high sequence identity with those of FMV isolates from Genbank, the most conserved being the RNA-3, with the RNA-3-encoded protein sharing 98–99% amino acids identity with its homolog of other isolates (Table 1). In general, at the genome level, the lowest nucleotide identity between FMV-Cic and other isolates was found in RNA-5 (84% at the nt level), whereas RNA-3 and RNA-4 showed the highest conservation levels, sharing 96% and 95% of nt identity with other isolates, respectively (Table 1).

### 3.2. RT-PCR Detection and Genetic Variability of FMV from Cyclamen Plants

RT-PCR assays conducted on symptomatic and asymptomatic cyclamen samples collected from two nurseries in southern Italy showed that four additional accessions (G, GU, TD, and AD) were infected with FMV. All positive accessions showed symptoms like those observed on Cic accession. Furthermore, results of PCR tests showed that three of these four FMV-infected accessions, i.e., GU, TD, and AD, were also infected by phytoplasma agents. Sequence analyses of these phytoplasma PCR amplicons showed that two phytoplasmas, belonging to two different groups, i.e., Aster yellows (16SrDNA I) and stolbur (16SrDNA XII), were present in these samples. However, when their sequences were compared with the homologous sequences present in the Genbank database, they were found to contain mutations suggesting that the isolates involved belong to new subgroups of Aster yellows and Stolbur. Further characterization of these two phytoplasmas is ongoing. Except for these infections, no other viruses or phytoplasma were found in the 18 collected samples.

The three primer pairs, specific to RNA-1, RNA-2, and RNA-3 of FMV, efficiently amplified partial cDNA fragments (RNA-1: 302 bp; RNA-2: 234 bp; RNA-3: 304 bp) from all four infected cyclamen accessions (G, GU, TD and AD). These fragments were sequenced in an effort to analyze the variability of cyclamen-infecting FMV isolates. This analysis showed nucleotide sequence identities between 86–99% for RNA-1, 93–99% for RNA-2 and 98–99% for RNA-3 with other FMV isolates from cyclamen (Supplementary Table S2). These identity levels decreased to 83% (GU vs. Tu-St50), 90% (TD and AD vs. Ca-Can01) and 93% (AD, TD, G, and GU vs. Ca-Can01) for RNA-1, -2, and -3, respectively, when fig isolates were included in the analysis (Supplementary Table S2). These results provide further confirmation that the highest level of sequence conservation is observed for the RNA-3, which encodes the nucleoprotein [21].

### 3.3. Phylogenetic Affinities of Cyclamen FMV Isolates

**Figure 2:**
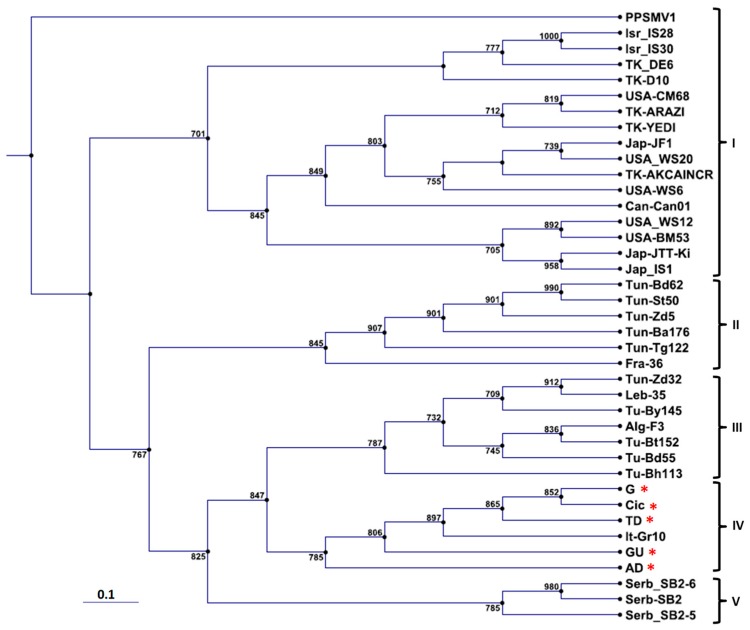
Phylogenetic tree constructed with the partial nucleotide sequences of RNA-3 (304 nt) of FMV isolates obtained in this study (labeled with an asterisk) and those retrieved from Genbank (32 isolates). Accession numbers of sequences used: AD (LS997754), G (LS997755), GU (LS997756), TD (LS997757), Cic (LT978307), D10 (KC182499), DE6 (KC182497), IS28 (KC182508), IS30 (KC182510), WS12 (KC1824789), JTT-Ki (AB697847), IS1 (AB697843), BM53 (KC182487), CD87 (KC182492), Can01 (HQ703345), Arazi (KC182500), YEDI (KC182501), CM68 (KC182490), WS6 (KC182476), AKCAINCR (KC182504), JF1 (AB697844), WS20 (KC182484), By145 (LN908832), 35 (LN908836), Zd32 (LN908827), Gr10 (FM991954), F3 (LN908837), Bt152 (LN908833), Bd55 (LN908829), Zd5 (LN908826), Ba176 (LN908834), 36 (LN908838), Tg122 (LN908831), Bd62 (LN908830), St50 (LN908828), SB2-5 (AB697855), SB2-6 (AB697856), and SB2 (AB697852). *Pigeonpea sterility mosaic emaravirus* 1 (HF568803) was used as out-species to root the tree. The tree was constructed from a multiple-sequence alignment generated with CLUSTAL W, using the “Neighbor Joining” (NJ) method in the software CLC Genomics Workbench. Numbers on branches indicate percentage of support out of 1000 bootstrap replications. Bootstrap values above 70% are shown. Scale bar represents 0.1 nt substitutions per site related to branch length. Alg (Algeria), Can (Canada), Fra (France), Ita (Italy), Isr (Israel), Jap (Japan), Leb (Lebanon), Serb (Serbia), Tk (Turkey), Tu (Tunisia), and the USA (United States).

The phylogenetic tree constructed with the partial RNA-3 nucleotide sequences of cyclamen isolates of FMV (TD, G, GU, AD, and Cic) and of the corresponding region of isolates from fig showed a five clades repartition. Clade I grouped the highest number of fig isolates from various origins, i.e., Israel and Turkey, the United States and Canada, and Japan, whereas clades II and III grouped mainly the Mediterranean fig isolates with some exceptions, i.e., some Tunisian, Israeli and Turkish fig isolates. FMV isolates from cyclamen formed a clade (IV) of their own with the Italian reference isolate from fig (Gr10) (Figure 2), whereas clade (V) grouped fig isolates from Serbia.

### 3.4. Etiology of FMV with Mosaic Disease on Cyclamen

All herbaceous hosts used in a mechanical transmission trial of the Cic and G cyclamen FMV isolates remained symptomless. Further RT-PCR testing confirmed the absence of FMV and of other cyclamen-infecting viruses from these inoculated plants. However, healthy leafed cyclamen stems grafted onto the Cic and the G accessions developed, within two months, symptoms of yellowing and mottling similar to those seen in these accessions (Figure 1).

## 4. Conclusions

The HTS technology has proven once more its superiority over other techniques in the identification of new plant pathogens in the absence of prior information on their nature or on their genome structure. Definitively, the outcome of this study, i.e., the identification of FMV in a new host (*Cyclamen persicum*), would not have been possible using more traditional investigation approaches. In addition, it is worth noting that through this technique it was possible to establish somehow the virological sanitary status of the Cic accession since no other viruses could be detected besides FMV. There is no absolute demonstration that the Cic accession is free from the presence of DNA viruses or viroids, as both are not efficiently detectable by HTS of dsRNA templates. However, the grafting trials which involved plants of the Cic and G accessions, that had been found free of other viral or phytoplasma agents by PCR, RT-PCR, and HTS approaches, strongly suggest that FMV is responsible for the observed leaf symptoms and flower breaking (Figure 1). This conclusion is further strengthened by the detection of FMV in the other three symptomatic cyclamen accessions and by its absence from all symptomless ones. The potential pathogenic contribution, if any, of the phytoplasmas detected in the GU, TD, and AD accessions is complicated to evaluate due to the simultaneous presence of FMV; therefore, it remains to be properly analyzed.

At the molecular level, the HTS approach greatly contributed to the determination of the complete sequencing of six RNA genomic segments of FMV, which were all found to have a highly similar structure with those of FMV isolates from fig, except for RNA-6, which is longer by one nucleotide. The phylogenetic grouping of FMV isolates from cyclamen together with an Italian fig isolate (Gr10) suggests that cyclamen infections probably occurred in the field. Whether these infections had occurred through the eriophyid mite *Aceria ficus*, notoriously known to transmit FMV in nature, and/or by another species common to fig and cyclamen, is yet to be determined. Certainly, the finding of FMV in cyclamen, the first non-fig host of this major fig virus, has important significance for the management of mosaic and mosaic-like diseases in fig and cyclamen, respectively.

## Figures and Tables

**Table 1:** Range of sequence identity matrix determined from nucleotides (nt) and deduced amino acids (aa) of each RNA segment of FMV-Cic and their homologues in other FMV isolates. n.a: not available. NCR: non-coding region. CR: coding region.

FMV-Cic	5′NCR %	CR %		3′NCR %	Isolate (Accession Number)
	nt	nt	aa	nt	Order of Isolates: JS1, SB1, Gr10, CAN01
**RNA-1**	84–100	88–97	93–96	87–99	(AB697826, AB697827, AM941711, HQ703343)
**RNA-2**	98–100	91–97	95–98	95–99	(AB697828, AB697829, FM864225, HQ703344)
**RNA-3**	93–99	96–98	98–99	90–96	(AB697843, AB697785, FM991954, HQ703345)
**RNA-4**	91–100	95–98	98–99	89–98	(AB697857, AB697865, FM992851, HQ703346)
**RNA-5**	84–100	84–97	82–96	84–95	(AB697871, AB697879, HE803826, n.a)
**RNA-6**	91–100	92–98	88–99	87–95	(AB697885, AB697893, HE803827, n.a)

## Supplementary Materials

The following are available online at http://www.mdpi.com/1999-4915/10/12/684/s1, Table S1: List of primers, designed on sequences of HTS-generated contigs, used in RT-PCR assays to amplify the full genomic RNA segments of FMV from Cic accession, Table S2: Sequence identity matrix determined from partial nucleotide sequences of RNA-1 (1), -2 (2) and -3 (3) segments of FMV from cyclamen and those from fig accessions present in the Genbank. Shadowed numbers represent the lowest identity found among different FMV isolates. Accession numbers of sequences used: RNA-1 (LN908800-LN908812, AB697827, AM941711, HQ703343), RNA-2 (LN908813-LN908825, AB697829, FM864225, HQ703344), RNA-3 (LN908826-LN908838, AB697785, FM991954, HQ703345).

## References

[1] Elbeaino T., Digiaro M., Alabdullah A.K., De Stradis A., Minafra A., Mielke N., Castellano M.A., Martelli G.P. (2009). A multipartite negative-sense single-stranded RNA virus is the putative agent of fig mosaic disease. J. Gen. Virol..

[2] Elbeaino T., Digiaro M., Mielke-Ehret N., Muehlbach H.P., Martelli G.P. (2018). ICTV Report Consortium. ICTV Virus Taxonomy Profile: Fimoviridae. J. Gen. Virol..

[3] Elbeaino T., Digiaro M., Martelli G.P. (2012). RNA-5 and -6, two additional negative-sense RNA segments associated with Fig mosaic virus. J. Plant Pathol..

[4] Elbeaino T., Digiaro M., Martelli G.P. (2009). Complete nucleotides sequence of four viral RNAs segments of fig mosaic virus. Arch. Virol..

[5] Slykhuis J.T., Gibbs A.J. (1973). Viruses and mites. Viruses and Invertebrates.

[6] Gentit P., Foissac X., Svanella-Dumas L., Peypelut M., Candresse T. (2001). Characterization of two different Apricot latent virus variants associated with peach asteroid spot and peach sooty riHTSpot diseases. Arch. Virol..

[7] Candresse T., Faure C., Theil S., Marais A. (2017). First report of Nectarine stem pitting-associated virus infecting *Prunus mume* in Japan. Plant Dis..

[8] Mielke N., Muehlbach H.P. (2007). A novel, multipartite, negative-strand RNA virus is associated with the riHTSpot disease of European mountain ash (Sorbus aucuparia L.). J. Gen. Virol..

[9] Foissac X., Svanella-Dumas L., Dulucq M.J., Candresse T., Gentit P. (2001). Polyvalent detection of fruit tree tricho, capillo and foveavirus by nested RT-PCR using degenerated and inosine containing primers (DOP RT-PCR). Acta Horticul..

[10] Mumford R.A., Barker I., Wood K.R. (1996). An improved method for the detection of tospoviruses using the polymerase chain reaction. J. Virol. Methods.

[11] Mumford R.A., Barker I., Wood K.R. (1994). The detection of Tomato spotted wilt virus using the polymerase chain reaction. J. Virol. Methods.

[12] Adkins S., Rosskopf E.N. (2002). Key West nightshade, a new experimental host for plant viruses. Plant Dis..

[13] Grieco F., Alkowni R., Saponari M., Savino V., Martelli G.P. (2000). Molecular detection of olive viruses. EPPO Bull..

[14] Peiman M., Xie C. (2006). Sensitive detection of potato viruses, PVX, PLRV and PVS, by RT-PCR in potato leaf and tuber. Australas. Plant Dis. Notes.

[15] Riga E., Larsen R., Eastwell K., Guerra N., Guerra L., Crosslin J.M. (2009). Rapid Detection of Tobacco rattle tobravirus in viruliferous paratrichodorus allius from greenhouse and field specimens. J. Nematol..

[16] Verma N., Kumar K., Kulshrestha S., Garg I.D., Raikhy G., Hallan V., Ram R., Zaidi A.A. (2006). Detection and Molecular Characterization of a Tomato Aspermy Virus Isolate Infecting Chrysanthemums in India. Acta Hortic..

[17] Davis R.E., Lee L.M. (1993). Cluster-specific polymerase chain reaction amplification of 16s rDNA sequences for detection and identification of mycoplasmaslike organisms. Phytopathology.

[18] Lee I.M., Gundersen D.E., Hammond R.W., Davis R.E. (1994). Use of mycoplasma-like organism (MLO) group-specific oligonucleotide primers for nested-PCR assays to detect mixed- MLO infections in a single host plant. Phytopathology.

[19] Gundersen D.E., Lee I.M. (1996). Ultrasensitive detection of phytoplasmas by nested-PCR assays using two universal primer pairs. Phytopathol. Mediterr..

[20] Ahrens U., Seemüller E. (1992). Detection of DNA of plant pathogenic mycoplasma-like organisms by a polymerase chain reaction that amplifies a sequence of the 16S rRNA gene. Phytopathology.

[21] El Air M., Mahfoudhi N., Dhouibi M.H., Digiaro M., Elbeaino T. (2016). Genetic variability of RNA-1, RNA-2 And RNA-3 of Fig mosaic virus isolates from Tunisia. J. Plant Pathol..

