# Supplementary material for: High-Throughput Sequencing Reveals Cyclamen persicum Mill. as a Natural Host for Fig Mosaic Virus
Source: Viruses. 2018 Dec 3;10(12):684. doi: 10.3390/v10120684 (PMC6316199; doi:10.3390/v10120684)
Supplement: Supplementary file 1 [file viruses-10-00684-s001.pdf]

Supplementary Materials:

Table S1. List of primers, designed on sequences of HTS-generated contigs, used in RT-PCR assays to amplify the full genomic RNA segments of FMV from Cic accession.

Primers	Sequence (5′ to 3′)	Nucleotide Position	Amplicon Size (bp)	Segment
R1-1s	AGTAGTGAACTCCCTTTATTAT	1-22	1057	RNA1
R1-1a	AATTGCAATCATTTAAGTACGT	1036-1057		
R1-2s	TATACCAAGATTGTTCAGATAG	953-974	1081	
R1-2a	GCCAAATGAATGGTTTAACAGC	2013-2034		
R1-3s	TCATGCAAATAAATTCCTGGGA	1911-1932	985	
R1-3a	CTGAATTTATAAATCTGATAAA	2875-2896		
R1-4s	AGCTACCAGATAATCTAGATTT	2801-2822	1222	
R1-4a	GAACAGTTCATTACCAACTATGA	4001-4023		
R1-5s	ATATTCAAACAGGTTTGATTGT	3918-3939	1224	
R1-5a	GCAATATCTTGAAGTTAAAAGG	5121-5142		
R1-6s	AGTGACAATGGCTAGACCATCA	5022-5043	1214	
R1-6a	ATCGGTGCAACATTAAACACTC	6215-6236		
R1-7s	GAGTTGAACCAACATCTTTATA	6140-6161	899	
R1-7a	AGTAGTGTTCTCCCTTTAATAT	7018-7039		
R2-1s	AGTAGTGAACTCCTCTTAAACAAAT	1-25	1250	RNA2
R2-1a	TAGGTGCACTTATAACAAAGGTT	1228-1250		
R2-2s	ATGTTTATGTTGGTCAAATCTGT	1155-1177	1097	
R2-2a	AGTAGTGTTCTCCTCTTAACATAAC	2051-2252		
R3-1s	AGTAGTGAACTCCCATAATTAACTC	1-25	890	RNA3
R3-1a	TTGCCTATAAGATTTAGGGCTT	869-890		
R3-2s	AGTATGACTGATGCTGATATTGT	787-809	703	
R3-2a	AGTAGTGTTCTCCCATAATTTAAAA	1466-1490		
R4-1s	AGTAGTGAACTCCTTACATCATAAA	1-25	774	RNA4
R4-1a	GCTTCCAGACATTAGAGCTACT	753-774		
R4-2s	ATGAAAAAGTTCATTGGGATGT	701-722	771	
R4-2a	AGTAGTGTTCTCCTTACATAACAAA	1351-1472		
R5-1s	AGTAGTGAACTCCCATAATTATAAT	1-25	849	RNA5
R5-1a	CTCTTTATTAAATCGAAGGCTA	828-849		
R5-2s	AGTCAAGCATCACAATCATATG	753-774	999	
R5-2a	AGTAGTGTTCTCCCATAACTAACTA	1728-1752		
R6-1s	AGTAGTGAACTCCCTATAAAACAAA	1-25	781	RNA6
R6-1a	TCGGTAATCTATATCTTAACTA	760-781		
R6-2s	ACATCTTCAGAACATATACATT	683-704	530	
R6-2a	AGTAGTGTTCTCCCTATAAGATAAA	1189-1213		

Table S2. Sequence identity matrix determined from partial nucleotide sequences of RNA-1 (1), -2 (2) and -3 (3) segments of FMV from cyclamen and those from fig accessions present in the Genbank. Shadowed numbers represent the lowest identity found among different FMV isolates. Accession numbers of sequences used: RNA-1 (LN908800-LN908812, AB697827, AM941711, HQ703343), RNA-2 (LN908813-LN908825, AB697829, FM864225, HQ703344), RNA-3 (LN908826-LN908838, AB697785, FM991954, HQ703345).

Isolates	AD	TD	G	Cic	GU	Tu BT152	Tu Bh113	Tu Bd55	Tu Zd32	Alg F3	Fr 36	Leb 35	It Gr10	Tu Tg122	Tu Zd5	Tu Ba176	Tu St50	Tu Bd62	Tu By145	Jap SB1	Ca can01
**AD** (1)	100																				
(2)																					
(3)																					
**TD** (1)	96	100																			
(2)	97																				
(3)	98																				
**G** (1)	89	91	100																		
(2)	93	94																			
(3)	99	99																			
**Cic** (1)	89	90	99	100																	
(2)	97	99	93																		
(3)	98	99	99																		
**GU** (1)	86	87	93	93	100																
(2)	93	94	96	93																	
(3)	99	99	99	99																	
**Tu-BT152** (1)	96	98	92	91	88	100															
(2)	93	93	96	93	96																
(3)	97	98	98	98	97																
**Tu-Bh113** (1)	96	98	92	91	88	99	100														
(2)	92	93	92	93	93	93															
(3)	96	97	97	97	96	98															

**Tu-Bd55** (1)	96	97	91	91	87	99	99	100						
(2)	93	95	97	94	97	98	94							
(3)	97	97	98	97	97	99	98							
**Tu-Zd32** (1)	96	97	91	91	88	99	99	98	100					
(2)	93	93	96	93	94	97	92	98						
(3)	97	98	98	98	98	99	98	98						
**Alg-F3** (1)	96	97	91	91	87	99	99	98	99	100				
(2)	93	94	96	93	96	99	93	99	98					
(3)	97	98	98	98	97	99	98	99	99					
**Fr-36** (1)	96	97	91	91	87	99	99	98	99	99	10			
(2)	93	93	94	93	94	96	93	97	96	96				
(3)	94	95	95	95	94	95	96	94	95	95				
**Leb-35** (1)	96	97	91	91	87	99	99	98	99	99	99	100		
(2)	94	96	95	95	95	96	95	97	95	96	95			
(3)	98	98	99	98	98	99	98	99	99	99	95			
**It-Gr10** (1)	95	97	92	91	88	98	98	98	98	98	98	98	100	
(2)	97	98	93	97	93	94	94	95	94	94	94	96		
(3)	99	99	99	99	98	98	97	98	98	98	95	99		
**Tu-Tg122** (1)	94	96	90	90	87	97	97	96	97	96	96	96	98	100
	92	93	92	93	93	93	99	94	92	93	93	95	94	
	96	96	97	96	96	96	97	95	96	96	97	96	97	

(2)																					
(3)																					
**Tu-Zd5** (1)	89	90	88	87	84	97	91	91	92	91	91	92	90	90	100						
(2)	93	94	93	93	94	94	93	95	93	94	95	95	94	93							
(3)	96	96	97	96	96	97	97	97	97	97	97	97	97	98							
**Tu-Ba176** (1)	91	92	89	89	86	94	94	93	94	94	94	94	93	91	96	100					
(2)	93	93	96	93	96	98	93	98	97	98	96	96	94	93	94						
(3)	95	96	96	96	95	96	97	95	96	96	96	96	96	97	98						
**Tu-St50** (1)	86	88	86	86	83	89	89	89	89	89	89	90	88	87	91	91	100				
(2)	94	94	94	94	95	94	95	96	95	95	95	96	95	95	96	94					
(3)	95	96	96	96	96	96	96	96	96	96	98	97	96	98	99	98					
**Tu-Bd62** (1)	89	90	89	88	85	92	92	91	92	92	92	92	91	90	94	93	97	100			
(2)	93	95	94	94	95	95	96	96	94	95	95	96	95	96	95	95	96				
(3)	95	96	96	96	96	96	96	96	96	96	97	97	96	97	98	98	99				
**Tu-By145**(1)	91	92	92	92	88	94	94	93	94	93	93	93	94	92	90	91	87	90	100		
(2)	93	94	96	93	96	98	93	99	98	99	96	96	94	93	94	99	95	95			
(3)	97	98	98	99	98	99	98	98	99	99	96	99	98	97	97	97	97	97			
**Jap-SB1** (1)	90	91	95	95	93	92	92	92	92	92	92	92	92	91	89	90	87	89	93	100	
(2)	94	96	96	95	96	96	93	97	95	96	95	97	95	93	94	96	95	96	96		
(3)	97	98	98	98	97	97	97	97	97	97	95	98	97	96	96	96	96	96	97		
**Ca-Can01**(1)	85	86	89	89	88	87	87	87	87	86	86	86	87	86	86	86	83	85	86	90	
(2)	90	90	92	91	91	90	89	92	91	91	90	92	90	89	89	91	90	91	91	94	100
(3)	93	93	93	94	93	93	94	93	93	93	93	94	93	94	94	94	94	93	94	95	
